# Practical approaches to automated digital image analysis of Ki-67 labeling index in 997 breast carcinomas and causes of discordance with visual assessment

**DOI:** 10.1371/journal.pone.0212309

**Published:** 2019-02-20

**Authors:** Ah-Young Kwon, Ha Young Park, Jiyeon Hyeon, Seok Jin Nam, Seok Won Kim, Jeong Eon Lee, Jong-Han Yu, Se Kyung Lee, Soo Youn Cho, Eun Yoon Cho

**Affiliations:** 1 Department of Pathology, CHA Bundang Medical Center, CHA University School of Medicine, Seongnam, Gyeonggi-do, Republic of Korea; 2 Department of Pathology, Busan Paik Hospital, Inje University College of Medicine, Busan, Republic of Korea; 3 Department of Pathology, Incheon St Mary’s hospital, College of Medicine, The Catholic University of Korea, Seoul, Republic of Korea; 4 Department of Surgery, Samsung Medical Center, Sungkyunkwan University School of Medicine, Seoul, Republic of Korea; 5 Department of Pathology, Samsung Medical Center, Sungkyunkwan University School of Medicine, Seoul, Republic of Korea; Columbia University, UNITED STATES

## Abstract

The Ki-67 labeling index (LI) is an important prognostic factor in breast carcinoma. The Ki-67 LI is traditionally calculated via unaided microscopic estimation; however, inter-observer and intra-observer variability and low reproducibility are problems with this visual assessment (VA) method. For more accurate assessment and better reproducibility with Ki-67 LI, digital image analysis was introduced recently. We used both VA and automated digital image analysis (ADIA) (Ventana Virtuoso image management software) to estimate Ki-67 LI for 997 cases of breast carcinoma, and compared VA and ADIA results. VA and ADIA were highly correlated (intraclass correlation coefficient 0.982, and Spearman’s correlation coefficient 0.966, p<0.05). We retrospectively analyzed cases with a greater than 5% difference between VA and ADIA results. The cause of these differences was: (1) tumor heterogeneity (98 cases, 56.0%), (2) VA interpretation error (32 cases, 18.3%), (3) misidentification of tumor cells (26 cases, 14.9%), (4) poor immunostaining or slide quality (16 cases, 9.1%), and (5) Estimation of non-tumor cells (3 cases, 1.7%). There were more discrepancies between VA and ADIA results in the group with a VA value of 10–20% compared to groups with <10% and ≥20%. Although ADIA is more accurate than VA, there are some limitations. Therefore, ADIA findings require confirmation by a pathologist.

## Introduction

Breast cancer is one of the most frequent malignancies in women. Many studies have sought to improve treatment outcomes for breast cancer, and molecular studies play a critical role in prognosis. Intensive molecular studies have made it possible to classify breast carcinomas, leading to improvements in treatment, prognosis prediction, and outcomes. Currently, the molecular classification of breast carcinomas can be easily confirmed according to estrogen receptor (ER), progesterone receptor (PR), human epidermal growth factor 2 (HER2), or Ki-67 labeling index (LI) status. Immunohistochemistry (IHC) is widely used to determine the expression of these markers. Among them, Ki-67 LI is a parameter for molecular classification and prognostic assessment[1–4]. For instance, in ER-positive and HER2-negative breast cancers in particular, the classification of subtypes is dependent on Ki-67 LI: tumors with low Ki-67 LI are classified into the luminal A group and those with high Ki-67 LI into the luminal B group[5]. Recurrence rate, prognosis, and therapeutic recommendations differ according to subtypes.

Traditionally, Ki-67 LI is estimated by visual observation. Despite its importance, Ki-67 LI has high inter-observer and/or intra-observer variability, and low reproducibility[6–8]. Several methods, such as a five-grade scale, have been suggested to resolve this problem[9–11]; however, low reproducibility remains an issue. Recently, computer-assisted image analysis has been used to achieve higher reproducibility of IHC results. Automated digital image analysis (ADIA) of Ki-67 LI in breast cancers obtains high quality data[12]. Several studies have observed ADIA to yield more reproducible and accurate measurement[13–16], and its application is being implemented in a clinical setting.

We performed visual assessment (VA) and ADIA simultaneously for breast carcinoma cases for approximately seven months. VA and ADIA were shown have their own strengths and weaknesses. In this study, we compared Ki-67 LI between by VA and by ADIA, and analyzed the causes of discrepancies. We sought to share the advantages and limitations of AIDA based on our experience.

## Materials and methods

### Patients and tissue specimens

This was a retrospective study conducted at a single institution. We collected all excised or biopsied specimens from patients diagnosed with breast carcinoma who underwent Ki-67 LI analysis at the Samsung Medical Center from December 2015 to June 2016. A total of 997 consecutive breast cancer specimens from 964 patients were obtained. All samples were formalin-fixed, paraffin-embedded and processed in a pathology laboratory according to standardized institutional protocols. Clinicopathological data were obtained by reviewing clinical charts for age, specimen type, stage, histological diagnosis, nuclear grade, IHC profiles for ER, PR and HER2, and the results of HER2 silver in situ hybridization (SISH). The study was approved by the institutional review board at Samsung Medical Center, Seoul, Korea (IRB No.2016-09-099), and informed consent was waived. All data were fully anonymized when we collected them.

### Immunohistochemical staining for Ki-67

IHC was performed with a Ventana automated immunostainer (Ventana, Tucson, AZ, USA). Tissue sections 4 μm thick were cut, dried, deparaffinized, rehydrated, and heated following a standard protocol. Primary Ki-67 antibody (MIB-1, DAKO, Denmark) was used at 1:200 dilution with the DAB detection system (Ventana) protocol.

### Assessment of Ki-67 by visual assessment

For VA, all Ki-67-immunostained slides were evaluated independently by two of four pathologists (AY Kwon, EY Cho, SY Cho, or HY Park) during routine reading. At the time of the evaluation, the pathologist was blinded to the previous estimated value. In cases of discrepancy between the two pathologists, an adjusted value was reached through consensus. Evaluation of Ki-67 nuclear positivity was calculated by unaided microscopic estimating to determine the percentage of tumor cells that were Ki-67 immunostaining-positive. If an invasive component was present, Ki-67 LI was estimated only in the invasive tumor component. If only an intraductal component was present, or the invasive area was less than 1 mm, Ki-67 LI was evaluated in the intraductal component. Using a protocol from the International Ki-67 in Breast Cancer Working Group[17], Ki-67 LI was evaluated in at least three fields at high-power magnification as the percentage of Ki-67 nuclear positivity. In the somewhat large tumor, we identified the homogeneity of Ki-67 staining at low-power magnification. In the homogeneously stained tumor, three randomly selected areas were used for evaluation. In the heterogeneously stained tumor, we evaluated the area including relatively hot spots, where the highest ratio of positive cells is shown. VA results for Ki-67 LI were estimated in 10% increments: if less than 10% was detected, it was subdivided into < 1%, < 5%, or 5–10%. The VA value used in this analysis was the median of the measurement range.

### Assessment of Ki-67 by automated digital image analysis

**Fig 1:**
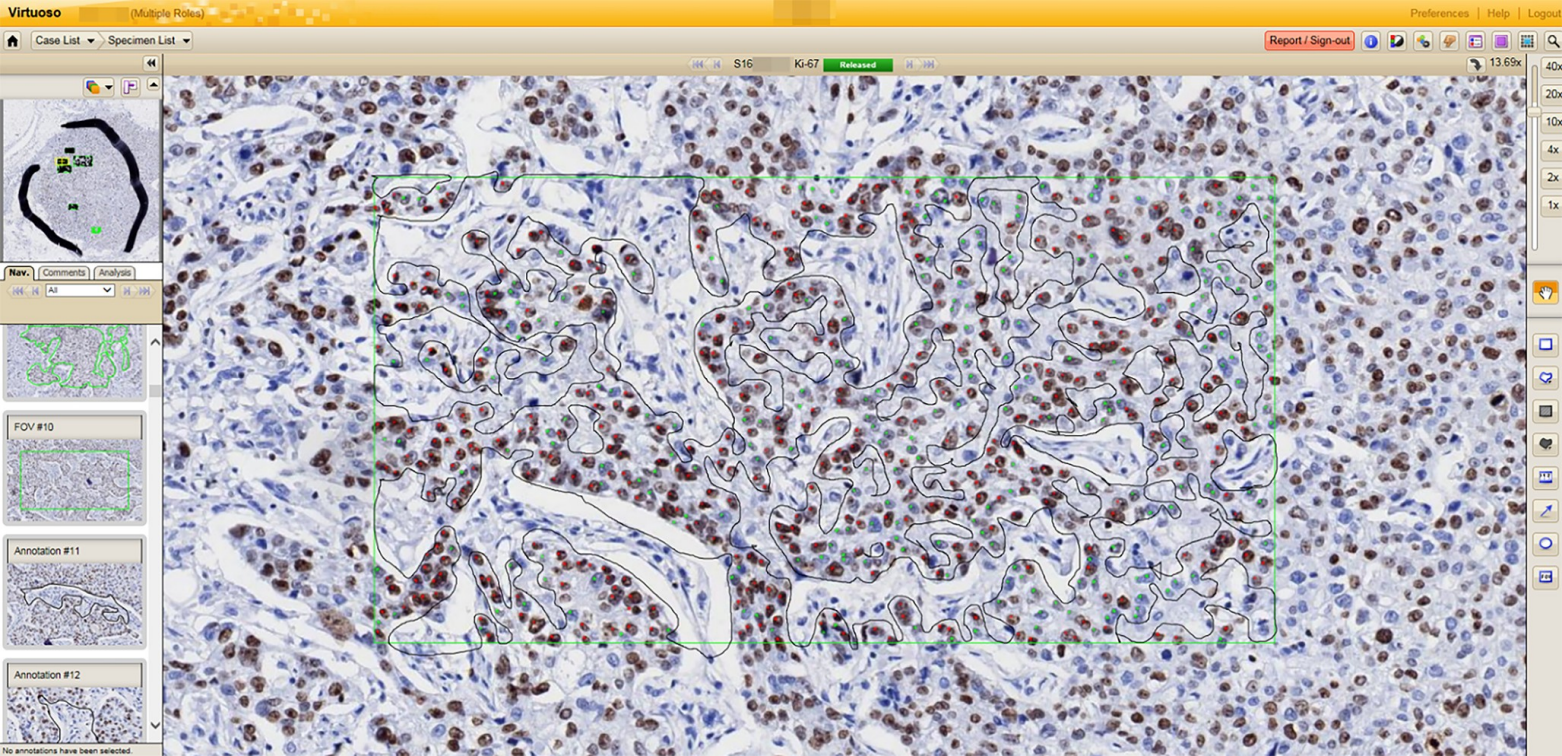
Automated digital image analysis by Ventana Virtuoso image analysis software. Each slide is scanned and several areas are selected. A green line surrounds the selected area, and a black line excluded area. The selected areas are analyzed automatically, marking stained tumor cells with red dots and non-stained tumor cells with green dots. The non-tumor cells are excluded in analysis automatically.

For ADIA, each Ki-67 stained slide of 997 cases was digitally scanned using a Ventana iScan (Ventana) with a 200x objective. Before scanning, pathologists selected a region of interest (ROI) about 25–50 mm^2^ in size (about one to two low-power field), including the area for VA assessment, especially hot spots. After scanning, the procedure, also called semi-manual image analysis, was performed as follows. Experienced physician assistants (PAs) selected some areas randomly in the ROI at 200x magnification. As seen in Fig 1, the PA-selected area is the green box. In this box, black lines were used to exclude the stromal area by the PA (Fig 1). After setting this area, Ventana Virtuoso image management software (ver.5.6, Ventana) was used to evaluate positive and negative stained tumor cells automatically. Ki-67-stained tumor nuclei were labeled and marked with DAB dye automatically by the software in the selected areas as red dots, while negative tumor nuclei remained the blue-color of hematoxylin staining, and were denoted by green dots. By repeating the above procedure, at least 1,000 tumor cells were evaluated according to recommendations of the Ventana software. In the practice, we tried to evaluated at least 1,500 tumor cells, an average of 3,000 cells. If tumor cells accounted for less than 1,000, all cells were evaluated. In ADIA, like VA, the invasive component was also assessed. When there was no invasive component or less than 1 mm, ADIA was evaluated in the carcinoma in situ. All ADIA results were stored on a server provided by Ventana. ADIA was performed by a PA and confirmed by a pathologist.

### Investigation of discrepancies between visual assessment and automated digital image analysis

We identified samples with a discrepancy between ADIA value and the median VA value greater than 5%, or an ADIA value out of the VA range. We sought to determine the cause of these discrepancies, and the limitations of VA or ADIA. By comparing Ki67-stained slides and the corresponding digital images, we surveyed and categorized the causes of these discrepancies.

### Statistical analysis

For analysis of the association between VA and ADIA, Pearson’s and Spearman’s rank correlations and intraclass correlation coefficient (ICC) were used. Correlations between clinicopathological parameters and differences in Ki-67 LI values were analyzed by chi-square test or Fisher’s exact test. Statistical analysis was performed using SPSS 16.0 for Windows (SPSS Inc., Chicago, IL, USA).

## Results

### Patient and tumor characteristics

We analyzed 997 breast carcinomas from 964 patients, with 22 patients having undergone bilateral mastectomy for bilateral breast carcinomas, and 11 patients undergoing Ki-67 LI testing of both a biopsy specimen of the breast or lymph node and a mastectomy specimen. The characteristics of 964 patients and 997 tumors are summarized in Table 1, and all data is provided in S1 File.

**Table 1:** Clinicopathologic characteristics of 997 cases with breast carcinoma of 964 patients.

Clinicopathologic parameters		n (%)
Age (years)	< 50	510 (52.9%)
	≥ 50	454 (47.1%)
Specimen	Core biopsy	14 (1.4%)
	Mammotome biopsy	8 (0.8%)
	Excision	27 (2.7%)
	Lumpectomy (partial mastectomy)	598 (60.0%)
	Total mastectomy	350 (35.1%)
T stage (11 cases, repeat exam) (10 cases, not assessable)	Tis	158 (16.2%)
	T1	506 (51.8%)
	T2	267 (28.3%)
	T3	42 (4.3%)
	T4	3 (0.3%)
N stage (11 cases, repeat exam) (65 cases, not assessable)	Metastasis present (N1, 2, 3)	280 (30.4%)
	Metastasis absent (N0)	641 (69.6%)
Histologic diagnosis (11 cases, repeat exam) (6 cases, not assessable)	Invasive ductal carcinoma	695 (70.9%)
	Ductal carcinoma in situ	149 (15.2%)
	Invasive lobular carcinoma	57 (5.8%)
	Lobular carcinoma in situ	6 (0.6%)
	Mixed invasive ductal and lobular carcinoma	8 (0.8%)
	Mucinous carcinoma	30 (3.1%)
	Metaplastic carcinoma	12 (1.2%)
	Invasive micropapillary carcinoma	9 (0.9%)
	Tubular carcinoma	5 (0.5%)
	Encapsulated papillary carcinoma	4 (0.4%)
	Solid papillary carcinoma	3 (0.3%)
	Giant cell carcinoma	2 (0.2%)
Nuclear grade (11 cases, repeat exam) (7 cases, not assessable)	Low	128 (13.1%)
	Intermediate	587 (59.9%)
	High	265 (27.0%)
Immuno-histochemical stain (11 cases, repeat exam)	ER positive	777 (78.8%)
	ER negative	209 (21.2%)
	PR positive	696 (70.6%)
	PR negative	290 (29.4%)
	HER2 positive	183 (18.6%)
	HER2 equivocal	39 (4.0%)
	HER2 negative	764 (77.5%)

Of the patients, 47.1% (454/964) were 50 years of age or older (23–85 years, median 49 years). Specimens were 14 core biopsies, 8 mammotome biopsies, 27 excisions, and 598 samples from partial mastectomy, and 350 from total mastectomy. Excluding 11 cases with repeat examination and 6 cases of exams where only metastatic lesions were examined, histological type was analyzed for a total of 980 breast carcinomas (Table 1). For invasive carcinomas, tumor size was 10 μm (microinvasive carcinoma with extensive intraductal component) to 14 cm (median, 1.7 cm). For 979 breast carcinomas of assessable nuclear grade, 13.1% were low nuclear grade, 59.9% were intermediate, and 27.1% were high. In addition, 78.8% of tumors were ER positive, 70.6% were PR positive, and 18.5% were HER2 positive. Samples from 60 patients (6.2%) were examined by IHC after neoadjuvant chemotherapy.

### Correlation between visual assessment and automated digital image analysis for Ki-67 LI

The median VA value from an experienced pathologist was 22.86% (0.5%–95.0%, median 15.00%) and the median ADIA value was 23.43% (0.09%–94.71%, median 15.51%). VA and ADIA evaluations were highly correlated (ICC 0.982, 95% confidence interval [CI] 0.980–0.984, Pearson’s correlation coefficient 0.982 [*p*<0.05], and Spearman’s correlation coefficient 0.966 [*p*<0.05]).

### Comparison of the differences between visual assessment and automated digital image analysis by the groups

The VA cutoff value of Ki-67 LI for molecular classification and decision making is 14%. Therefore, we classified all cases according to the VA value of Ki-67 LI as <10%, 10%-20%, and ≥20%. The 10%-20% and <10% (ICC 0.407 and 0.581, respectively) groups showed poorer consistency than the of ≥20% group (ICC 0.947, Table 2)

**Table 2:** The intraclass correlation coefficient (ICC) and Spearman’s correlation coefficient, stratified by visual assessment values.

Groups	n	ICC (95% CI)	p-value	Spearman’s ρ	p-value
<10%	350	0.581 (0.507–0.646)	<0.001	0.698	<0.001
10%-20%	264	0.407 (0.301–0.503)	<0.001	0.546	<0.001
≥20%	383	0.965 (0.957–0.971)	<0.001	0.947	<0.001

### Cause of discrepancies between visual assessment and automated digital image analysis

We retrospectively analyzed 175 cases with discrepancies greater than 5% between the median VA value of VA and ADIA results or with ADIA values beyond the VA range.

Causes of discrepancies were as follows: (1) tumor heterogeneity (98 cases, 56.0%); (2) VA interpretation error (32 cases, 18.3%); (3) misidentification of tumor cells (26 cases, 14.9%); (4) poor quality immunostaining or mounting, including weak staining, cytoplasmic staining, or problems with fixation or paraffin block-cutting (16 cases, 9.1%); and (5) non-tumor cells such as inflammatory cells and/or stromal cells included in estimation (3 cases, 1.7%). Analysis of causes of errors is summarized in Table 3.

**Table 3:** Causes of Ki-67 LI discrepancies between visual assessment and automated digital image analysis.

Cause of discrepancy	n (%)
Tumor heterogeneity	98 (56.0%)
Visual assessment interpretation error	32 (18.3%)
Misidentification of tumor cells	26 (14.9%)
Poor immunostaining or slide quality	16 (9.1%)
Non-tumor cells estimated	3 (1.7%)

### Statistical analysis of discrepancies and clinicopathological parameters

We examined correlations according to type of specimen (biopsy, excision, mammotome, and mastectomy), histological subtype, tumor size, microinvasiveness, nuclear grade, neoadjuvant chemotherapy, PA who performed ADIA, or pathologist who performed VA. Biopsied specimens showed significantly larger discrepancies between VA and ADIA than mastectomy specimens (p = 0.035). Also, intermediate nuclear grade tumors had significantly larger discrepancies between VA and ADIA than tumors of high (p = 0.018) and low nuclear grade (p = 0.048). Other parameters were not correlated with discrepancies in Ki-67 LI.

## Discussion

Ki-67 LI is an important marker for classification, treatment planning and prognosis of various malignant tumors. In breast carcinoma, as in other carcinomas, high Ki-67 LI is associated with worse outcomes[6, 18]. Although Ki-67 LI is important clinically, traditional evaluation depends on subjective assessment by pathologists. Relatively inaccurate and low-reproducible VA indexes[19] can affect the association between Ki-67 index and prognosis. ADIA is one solution for accurate and reproducible assessment of Ki-67 LI, that was recently introduced[16]. Using ADIA, relatively accurate estimation of stained tumor cells is possible in a few seconds. Permanent digital data is generated and repeat assessment is available at any time. ADIA offers little fear of loss of staining, labeling or whole slides. We used ADIA for breast cancer evaluation for about seven months. Through that experience, we recognized the practical limitations of ADIA use.

Previous studies focused mainly on correlation between VA and ADIA results, including area-setting methods[20, 21], ROI size and the number of cells[22], dual staining with cytokeratin and Ki-67[14], and other applications[23–25]. However, in this study, we investigated the limitations of ADIA in practice as well as comparing VA and ADIA, and we recommended methods to overcome the potential problems. We analyzed 175 cases with some discrepancies between VA and ADIA results.

Correlation analysis showed high concordance between VA and ADIA values. This result may indicate consistency; however, considering the effort to develop and expand ADIA use, it may be disappointing. Previous published studies showed good concordance between VA and ADIA, not only in breast cancer[26, 27], but also in other cancers[28–30]. Therefore, ADIA’s main advantages are reproducibility[15, 16], permanence, and data storage. Also, ADIA is useful when it required specific values; for example, “14%” of breast cancers.

**Fig 2:**
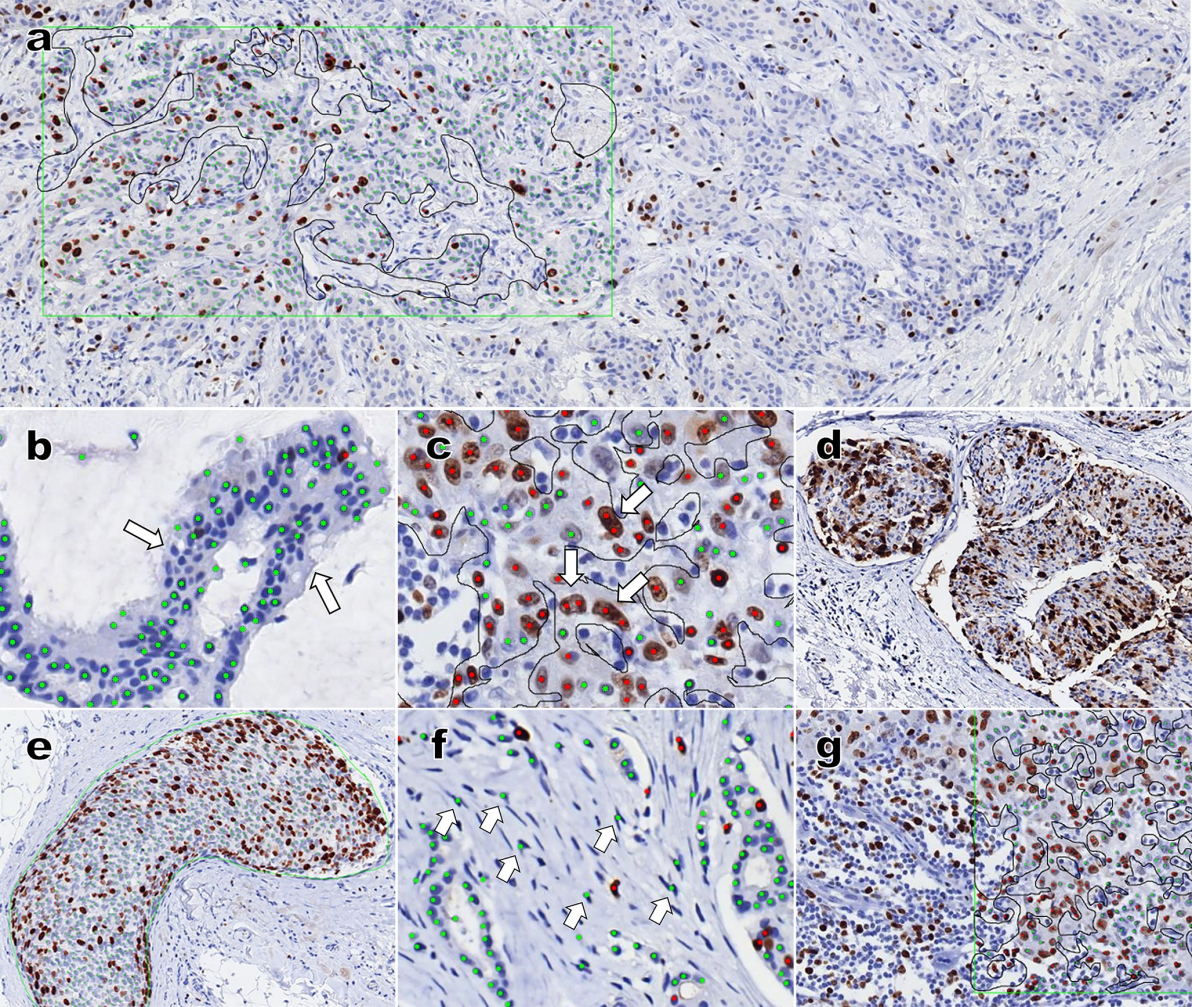
The causes of discrepancy between two visual assessment and automated data image analysis. (a) Heterogeneity of Ki-67 immunostaining. The left side green-lined box had a higher Ki-67 labeling index (LI) than the right side, indicating a difference between visual assessment and automated digital image analysis. (b—c) Misidentification of tumor cells. (b) Some tumor cells (arrow) were not recognized due to limitation of the automated algorithm. (c) Pleomorphic large cells or dumbbell-shaped tumor cells were estimated as two or more cells. (d—e) Poor Ki-67 immunostaining quality. (d) Poor tissue fixation led to poor Ki-67 staining quality and interfered with automatic image analysis. (e) Changing the tissue block and re-staining for Ki-67 led to clear staining and image analysis. (f—g) Erroneous recognition of non-tumor cells as Ki-67-negative tumor cells. (f) Some stromal cells were assessed as Ki-67-negative tumor cells. (g) Areas with stromal cells and/or inflammatory cells should be excluded; tumor cells were selected (black line) to exclude non-tumor cells as much as possible.

Through our experience and analysis of 175 cases, we identified the following possible problems. First, heterogeneity is the most frequent cause of discordance between VA and ADIA. An example of heterogeneity is presented in Fig 2A. Whereas pathologists intuitively judged the wider area during VA, the ADIA area was smaller.

Heterogeneity of Ki-67 labeling is an ongoing issue. In a study by Koopman et al.[31], heterogeneity was also the most common reason for discrepancies between manual counting and ADIA. To overcome heterogeneity, it is commonly recommended that Ki-67 LI be assessed in peripheral tumor areas with higher LI; however, area selection remains the most important aspect of assessment of Ki-67 LI. Some studies have worked to address this problem, by investigating selection method, ROI size, and the number of cells. Ki-67 LI is dependent on ROI size, and the number of cells estimated, which is a critically important parameter in Ki-67 quantification by ADIA. Christgen et al. showed that median Ki-67 LI decreased from 55 to 15% depending on ROI size and suggested that a minimal ROI of 500 cells is an acceptable compromise[22]. Our findings showing that biopsied specimen analysis yielded greater discrepancies between VA and ADIA results than analysis of mastectomy specimens support Christgen et al.’s results. Because biopsied specimens usually have relatively few tumor cells that are interrupted by stroma, smaller numbers of tumor cells are counted and discrepancies between VA and ADIA may be more prevalent. Analyzing the entire slide may be a way to resolve the heterogeneity-problem. Laurinavicius et al. assessed Ki-67 expression on whole-slide images by digital image analysis and demonstrated that Ki-67 is an independent prognostic indicator of overall survival[32]. Zhong et al. investigated an average score method, assessing at least three selected areas on an entire digital slide, with comparison to a hot-spot score method focusing on hot spot areas[33]. This average score method led to slightly better agreement between VA and ADIA results than the hot-spot score method, but differences were extremely small. Whether values from whole-slide or hot-spot analysis by ADIA are more useful for prognosis remains unclear. Further studies analyzing ADIA results and patient prognosis are needed to improve the precision of scoring and to develop new cutoff values for Ki-67 LI for breast cancer subtyping. The area for ADIA was selected in which was previously assessed by VA, and estimated at least 1,500 and an average of 3,000 tumor cells by ADIA; however, heterogeneity remains a problem in ADIA. Area selection is always accompanied by selection bias unless a whole slide section is measured. Therefore, in practice, we recommend that the basic protocol for selecting areas is followed, and that a large number of tumor cells, at least 1,000 cells, are counted. For cases with extremely heterogeneous staining, pathologists can confirm the findings using VA. Pathologists need to carefully consider ROI selection, and may try to estimate more areas multiple times with ADIA.

The second most common cause of discrepancy was VA interpretation error. We were aware of interpretation errors at the time of glass slide review. The main reason for errors was intuitive or partial measurements, or mistakes in recording. In these 32 cases, we were able to use ADIA to correct human error.

The third cause of differences was misidentification of tumor cells. In some cases, Ki-67-negative tumor cells were not marked (Fig 2B). This might have been due to weak hematoxylin staining and/or a limitation of the automated software algorithm. Another possibility in a few cases was a pleomorphic, large cell or a dumbbell-shaped tumor cell being estimated as two or more cells (Fig 2C). When we recognized these errors during confirmation, we requested that PAs repeat ADIA. Therefore, pathologists must be verified for Ventana scanning images and judging if ADIA analysis is appropriate. If needed, re-analysis of other areas must be performed. We think that manual marking or deleting dots from images may be helpful; however, this is currently not available in Ventana software, which must follow the algorithm provided.

The fourth cause of discrepancies was poor Ki-67 immunostaining quality. Problems with tissue fixation, tissue processing, and/or thick slices because of abundant fat tissue, were included in this category. The spindling morphology of tumor cells due to poor preparation or fixation could not be used to differentiate tumor cells from stromal cells on ADIA (Fig 2D). A few samples showed clearing of nuclei or cytoplasmic Ki-67-staining, which interfered with automatic image analysis. Through experience we realized that these slides made ADIA analysis difficult. We began to evaluate slide quality during VA, and re-stained for Ki-67, recut slides, or changed tissue blocks as necessary (Fig 2E). There is still the potential for error due to these issues, and pathologists and PAs should be fully aware of these concerns.

Another cause of discrepancy was misidentification of non-tumor cells as Ki-67-negative tumor cells. ADIA can recognize stromal cells and/or inflammatory cells as tumor cells, so they are assessed and counted as Ki-67-negative tumor cells (Fig 2F). Therefore, it was helpful that non-tumor cells were excluded in the estimated area as much as possible, by using the black line in Fig 2G. However, non-tumor cells, such as lymphocytes infiltrated in carcinomas, were unavoidably included in some estimates. Harvey et al. and Roge et al.[14, 34] suggested double-staining of cytokeratin and Ki-67 to exclude stromal and/or inflammatory cells. The estimation protocol we used, Ventana Virtuoso image management software, did not involve double-staining. We expect that double-staining could be helpful, but only 1.7% of our cases involved this error. In other words, while this problem has the potential to affect Ki-67 LI measurement, it was not found to be a major concern in our protocol. A new measurement method using double-staining could overcome this issue, enabling more accurate measurements.

In the situation where the discrepancy between VA and ADIA was more than 5%, ADIA tended to measure relatively higher than VA; 116 cases showed higher Ki-67 LI in ADIA and 59 cases in VA. We analyzed the relative values of VA and ADIA for each cause. In cases of heterogeneity, 69 cases (70.4%) showed higher ADIA than VA, indicating a tendency toward relatively high Ki-67 LI on ADIA when there was heterogeneity. The smaller and more compact the ROI, the more Ki-67 LI was expected to increase. In cases of VA error, VA was higher in half cases (16/32, 50.0%) and half cases showed higher ADIA. In cases of misidentification of tumor cells, there were 22 cases (84.6%) with higher ADIA values and 4 cases (15.4%) with higher VA values. It is believed that tumor cells without Ki-67 labeling were often not counted, or stained tumor cells were counted multiple times. In the cases of poor slide quality, half (8/16, 50.0%) showed higher VA values and the other half showed higher ADIA values. The three cases of non-tumor cell estimation consisted of 2 cases with higher VA and 1 case with higher ADIA.

Because 14% is widely used as a cutoff for molecular classification and a prognostic factor[35], we employed three classification groups, <10%, 10%-20%, and ≥20%, stratified by VA values. We compared the concordance between VA and ADIA among the three groups. As with previous studies[33], the intermediate Ki-67 LI group (10%-20%) showed relatively weak concordance between VA and ADIA. In addition, we analyzed ADIA values of samples with 10%-20% of VA Ki-67 LI, which included 198 cases (19.9%). Additionally, we selected the cases that were ER/PR positive and HER2 negative, because Ki67 LI is an important factor in luminal A and B subtyping. A total of 560 cases were culled. Of these, in 4 cases, the VA value was 5%-15%, but ADIA values were over 14% higher; in other words, according to the VA values, the cancers were luminal A subtype, but according to ADIA values, they were luminal B subtype. When the VA value was 10%-20%, there were more luminal B subtypes (Ki-67 LI≥14%, 91/131 cases) than luminal A subtypes (Ki-67 LI<14%, 40/131 cases). Thus, when the VA value is close to 14%, there is weak concordance, and it is not easy to determine the subtype. In these cases, ADIA will be certainly helpful. Even when using ADIA, there may still be cases for which classification is difficult. We recommend repeating measurement using more cell counts or a bigger area, and further studies on prognosis and subtype classification according to new measurement values are needed.

Carcinomas with intermediate nuclear grade showed larger differences in Ki-67 LI than carcinomas with low and high nuclear grades. We propose that high-grade nuclei were easily recognized due to their bigger size on both VA and ADIA. Also, high-grade tumors usually showed dense nesting patterns of tumor cells without accompanying stroma, with low overall heterogeneity because Ki-67 was diffusely stained. In low-grade tumors, Ki-67 LI was usually estimated to be less than 5% or 10%. Therefore, the discrepancy between VA and ADIA was not large among low-grade tumors.

VA has well-known limitations, especially inter-observer and intra-observer variability. Ki-67 LI by ADIA is expected to yield more reproducible and accurate values, and ADIA was a useful method in our experience for seven months of experience. Some limitations of ADIA were established from that experience, and as well as retrospective analysis of discrepancies between VA and ADIA values. In this paper, we analyzed the results of ADIA, and possible problems associated with the use of ADIA or VA. We sought to overcome these limitations, and have succeeded to some extent. We realized that final confirmation of ADIA data by experts was essential. These recommendations should lead to more accurate Ki-67 LI measurement, and these data will support prognostic analysis, which in turn will be helpful in tumor classification, determining prognosis, and treatment planning.

## Supporting information

S1 File Clinicopathologic characteristics of 997 cases with breast carcinoma of 964 patients, with VA and ADIA values. (XLSX)
